# Genome Sequencing of an Extended Series of NDM-Producing Klebsiella pneumoniae Isolates from Neonatal Infections in a Nepali Hospital Characterizes the Extent of Community- versus Hospital-Associated Transmission in an Endemic Setting

**DOI:** 10.1128/AAC.03900-14

**Published:** 2014-12

**Authors:** N. Stoesser, A. Giess, E. M. Batty, A. E. Sheppard, A. S. Walker, D. J. Wilson, X. Didelot, A. Bashir, R. Sebra, A. Kasarskis, B. Sthapit, M. Shakya, D. Kelly, A. J. Pollard, T. E. A. Peto, D. W. Crook, P. Donnelly, S. Thorson, P. Amatya, S. Joshi

**Affiliations:** a Nuffield Department of Clinical Medicine, University of Oxford, Oxford, United Kingdom; b Wellcome Trust Center for Human Genetics, Oxford, United Kingdom; c NIHR Biomedical Research Center, University of Oxford/Oxford University Hospitals NHS Trust, Oxford, United Kingdom; d School of Public Health, Imperial College London, London, United Kingdom; e Icahn School of Medicine at Mount Sinai, New York, New York, USA; f Department of Pediatrics, Patan Hospital, Kathmandu, Nepal; g Oxford Vaccine Group, Center for Clinical Vaccinology and Tropical Medicine, University of Oxford, Oxford, United Kingdom

## Abstract

NDM-producing Klebsiella pneumoniae strains represent major clinical and infection control challenges, particularly in resource-limited settings with high rates of antimicrobial resistance. Determining whether transmission occurs at a gene, plasmid, or bacterial strain level and within hospital and/or the community has implications for monitoring and controlling spread. Whole-genome sequencing (WGS) is the highest-resolution typing method available for transmission epidemiology. We sequenced carbapenem-resistant K. pneumoniae isolates from 26 individuals involved in several infection case clusters in a Nepali neonatal unit and 68 other clinical Gram-negative isolates from a similar time frame, using Illumina and PacBio technologies. Within-outbreak chromosomal and closed-plasmid structures were generated and used as data set-specific references. Three temporally separated case clusters were caused by a single NDM K. pneumoniae strain with a conserved set of four plasmids, one being a 304,526-bp plasmid carrying *bla*_NDM-1_. The plasmids contained a large number of antimicrobial/heavy metal resistance and plasmid maintenance genes, which may have explained their persistence. No obvious environmental/human reservoir was found. There was no evidence of transmission of outbreak plasmids to other Gram-negative clinical isolates, although *bla*_NDM_ variants were present in other isolates in different genetic contexts. WGS can effectively define complex antimicrobial resistance epidemiology. Wider sampling frames are required to contextualize outbreaks. Infection control may be effective in terminating outbreaks caused by particular strains, even in areas with widespread resistance, although this study could not demonstrate evidence supporting specific interventions. Larger, detailed studies are needed to characterize resistance genes, vectors, and host strains involved in disease, to enable effective intervention.

## INTRODUCTION

Klebsiella pneumoniae is a major pathogen, especially in neonatal critical care (1), and in association with extended-spectrum beta-lactamases (ESBLs) and/or carbapenemases, it results in increased hospital costs, longer stays, and high rates of mortality (2, 3). Furthermore, the success of controlling outbreaks of carbapenemase-producing Klebsiella spp. is mixed (4, 5). In resource-limited settings, such as South Asia, multidrug-resistant Enterobacteriaceae are widespread (1, 6), and therefore, delineating the relative contributions of within-hospital transmission of resistant strains and mobile genetic elements versus recurrent importation of these from the community is challenging.

Carbapenem resistance in Klebsiella spp. is typically caused by hydrolytic enzymes or nonexpression of porin genes/increased efflux pump activity in the presence of ESBLs (7). Of particular concern is the recent emergence of transmissible carbapenemases, such as Klebsiella pneumoniae carbapenemase (*bla*_KPC_) (8) and New Delhi metallo-beta-lactamase (*bla*_NDM_) (9). These genes are widely transmitted intra- and interspecies on mobile genetic elements. For example, *bla*_NDM_ has been observed in at least 40 countries, on different plasmids, in several species of Enterobacteriaceae (10), and in environmental and animal samples (11). The reported prevalence likely underrepresents the problem, as screening frequently relies on insensitive phenotypic methods; molecular methods are more robust (12).

Whole-genome sequencing (WGS) (13–15) can markedly alter classical interpretations of transmission (14, 15). Several studies have used lower-resolution typing methods, such as pulsed-field gel electrophoresis (PFGE), to characterize outbreaks caused by carbapenem-resistant Klebsiella spp., and two published studies have successfully utilized WGS, describing KPC- and NDM-1-producing strains, respectively (16, 17). The former study described a 6-month outbreak (22 isolates) using genetic and epidemiological data, supporting both patient-to-patient and ventilator-to-patient transmission. The latter study was smaller (8 isolates) and revealed the likely transmission of NDM K. pneumoniae at a U.S. hospital over 4 months.

To extend the results of previously published work, we used Illumina and PacBio SMRT (single-molecule real-time sequencing) technology (Pacific Biosciences) to recover nearly complete genetic information, including that of episomal structures, of a series of disease-causing, multidrug-resistant K. pneumoniae isolates collected in a hospital neonatal service in Nepal. In addition, we made genetic comparisons with other contemporaneously collected clinical isolates (both K. pneumoniae and other Gram-negative bacilli), thereby investigating the hypothesis that these drug-resistant cases were occurring as a result of independent introductions of strains/plasmids from the wider community.

## MATERIALS AND METHODS

Patan hospital is a 450-bed hospital in Kathmandu, Nepal, with a pediatric unit handling ∼9,500 live births, 35,000 outpatient visits, and 2,600 admissions/year. Neonatal care is managed in two nurseries, one “clean,” for non-sepsis-related supportive care, and one “septic,” for patients who have major risk factors for or a diagnosis of sepsis. The neonatal and pediatric intensive care units (NICU and PICU) are located close to the nurseries.

The suspected first infected neonatal case was a low-birth-weight premature baby born in the hospital on 9 August 2011. Postnatally, he was admitted to the NICU for respiratory distress. Early blood cultures were negative, but on day 8, K. pneumoniae was cultured, initially susceptible only to carbapenems. The child died 4 days later, despite treatment.

Cases of sepsis associated with similar drug-resistant K. pneumoniae-positive cultures susceptible only to one or more of the carbapenems, colistin, or tigecycline were defined as part of an epidemiological cluster, with the last observed case on 30 June 2012. To contextualize genetic variation in cluster isolates and identify wider spread of resistance genes/elements, we randomly sampled a subset of stored Enterobacteriaceae and other Gram-negative bacilli, stratified by susceptibility profile, age (adults/children), and hospital-associated (sampled ≥48 h after admission) versus community-associated (sampled <48 h after admission, with no previous admission to Patan hospital and delivery outside a health care facility) infections (see Section 1 in the supplemental material).

Infection control was enhanced from December 2011, including changes in cleaning, equipment, and surveillance protocols and closure of the original clean nursery (see Section 2 in the supplemental material).

### Laboratory and sequencing methods.

All samples were originally processed locally. Species identification was based on biochemical profiling. Antimicrobial susceptibility was assayed using the disk diffusion method (18) and was reassessed using broth microdilution in Oxford (BD Phoenix automated microbiology system; Oxford, United Kingdom).

Environmental sampling is performed routinely as part of infection control surveillance, and this included the study period. Typically, paired swab samples are collected at 2-week intervals before and after intensive cleaning from five different randomly selected sites in the NICU and the established clean and septic nurseries, including laryngoscope blades, ventilators, stethoscopes, equipment trolleys, incubators, central lines, endotracheal/suction tubes, tap water, floors, and door handles. If a patient was considered part of a K. pneumoniae case cluster, a set of these surveillance swabs was taken from the patient's incubator or bed. Rectal swab samples were taken from all nursing and medical staff in the neonatal nurseries, NICU, and PICU (between 9 and 11 July 2012). Samples were also collected from purified water used for clinical purposes (26 September 2011), disinfectants (13 December 2011), and air conditioners in the nurseries and ICUs (12 December 2011). All samples were plated on MacConkey agar and incubated aerobically; Klebsiella isolates were identified as described above.

DNA was extracted from subcultured frozen isolate stocks (QuickGene; Fujifilm, Tokyo, Japan) with a mechanical lysis step (FastPrep; MP Biomedicals, Santa Ana, CA). All extracts were sequenced on the HiSeq 2000 platform (Illumina, San Diego, CA), generating 100-base paired-end reads. For the isolate cultured from the first infected neonatal case (PMK1), PacBio SMRT sequencing was also undertaken.

### Sequence data analysis.

Species identification was confirmed with Kraken (19), and Illumina reads were mapped to species-specific reference genomes (see Sections 3 and 4 in the supplemental material). Mapping/variant-calling methodologies were performed as previously described (14). Reads were also *de novo* assembled using Velvet and VelvetOptimizer (20, 21); BLASTn was used to identify the presence/absence of resistance gene variants in assemblies (22) and infer multilocus sequence types (STs) (23).

A maximum-likelihood phylogeny of the first K. pneumoniae isolate per case based on single-nucleotide variants (SNVs) distributed over the core mapped genome was constructed in PhyML (see Section 5 in the supplemental material) (24).

The PacBio reads for PMK1 were assembled with HGAP (hierarchical genome assembly process) and Quiver (25). Chromosomal and plasmid contigs were identified using BLASTn (26) against the NCBI nucleotide sequence database. Circular overlaps in plasmid contigs were identified with nucmer (27). Reference plasmid sequences were closed and corrected with visual inspection of BWA (Burrows-Wheeler aligner)-generated Illumina and PacBio read alignments to plasmid contigs (28) and annotated with Prokka (29).

All strains were mapped to the PacBio-generated reference sequence (chromosome plus plasmids) with BWA. Structural variation in plasmids was identified by plotting the mean read coverage for each 1,000 bp of the reference sequence divided by the mean read coverage across the whole reference sequence, capped to a maximum of one. Precise breakpoints were identified from the inspection of mapped reads. The presumptive plasmid structures generated are denoted pPMK[isolate number]-[reference plasmid suffix], e.g., pPMK17-NDM. The NDM gene copy number was estimated by read counts of the NDM regions divided by the total number of reads, scaled to the count for pPMK1-NDM, and then rounded to the nearest whole number.

The genetic outbreak was then defined as any cases from the epidemiological cluster and closely genetically related isolates forming a distinct group on the maximum-likelihood tree. Comparing these to the PacBio-generated reference chromosome, a mutation rate for the outbreak strains was estimated using a time-scaled analysis in BEAST (see Section 6 in the supplemental material) (30), including longitudinal isolates from individuals. The most likely transmission chain was inferred using the R Outbreaker package (31) (see Section 7 in the supplemental material).

### Nucleotide sequence accession numbers.

Illumina-generated sequence data for the whole data set have been deposited at NCBI (project accession number PRJNA253300) (see Section 1 in the supplemental material). Sequence data for the PacBio/Illumina-generated PMK1 reference chromosome and plasmids have been deposited in GenBank with accession numbers as follows: (i) PMK1 (chromosome), CP008929; (ii) pPMK1-A, CP008930; (iii) pPMK1-B, CP008931; (iv) pPMK1-C, CP008932; and (v) pPMK-NDM, CP008933. Sequence data were also deposited in the Sequence Read Archive under accession number SRP043586.

## RESULTS

**FIG 1:**
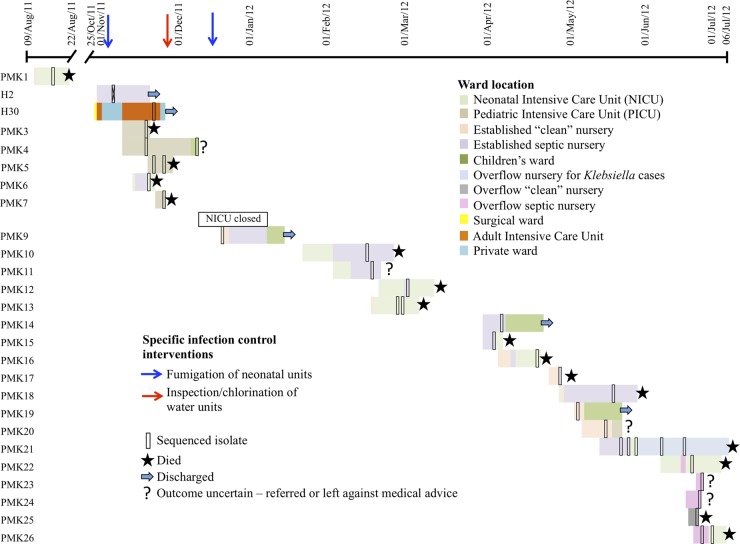
Timeline of Klebsiella pneumoniae cases, including individuals who were both part of epidemiologically defined case clusters and had genetically linked outbreak strains. H2 was found to be genetically unrelated to the other outbreak isolates; H30 was thought to share a relatively recent common ancestor but was not involved in the immediate transmission network. There were no clinical details available for H1460, which is therefore not shown.

Of 102 strains sequenced, 55 were confirmed as K. pneumoniae, 43 as other Enterobacteriaceae, and 4 as other Gram-negative bacilli. Of 55 K. pneumoniae isolates sequenced from 47 individuals, 34 isolates sampled from 26 individuals were part of the genetically defined outbreak. All outbreak isolates were cultured from blood, except for PMK9, which was cultured from cerebrospinal fluid. Figure 1 shows the timeline of cases in the epidemiologically defined cluster and/or the genetically defined outbreak. The prolonged time intervals between some cases (PMK1 to PMK3, 96 days; PMK9 to PMK10, 57 days; and PMK12 to PMK15, 34 days) demonstrate the challenge of distinguishing between multiple importations and ongoing spread. The outbreak affected all units, including the overflow nurseries established toward the end of the outbreak in a different hospital building.

The outbreak-associated mortality was high, with 16 (64%) inpatient deaths in 25 neonates (6 had an unknown outcome, e.g., referred elsewhere or left against medical advice), in contrast with a hospital-wide contemporaneous neonatal death rate of 46/6,908 (0.7%). The neonatal critical care mortality rates were 46% (45/98 cases) during the outbreak period and 27% (32/117) in the year following the final case (*P* = 0.007, Fisher's exact test).

Approximately 30 sites per month were sampled from the three neonatal units surveyed; the heaviest environmental contamination with Klebsiella spp. was observed at the onset of the outbreak, between August 2011 and January 2012 (mean of 2 environmental swabs positive [range, 0 to 3]), as opposed to the preceding and subsequent 6-month periods (no sites positive at any time point; mean of 0 sites positive [range, 0 to 1], respectively) (see Section 8 in the supplemental material). Klebsiella spp. bacteria were isolated from a number of environmental cultures, including samples obtained from laryngoscope blades, suctioning equipment, purified water containers, tap water, soap dispensers, a burette set surface swab, and a health care worker's hands; none of these underwent susceptibility testing. Eighteen of 69 (26%) rectal swab samples cultured Klebsiella spp., but none were ESBL or carbapenemase producers. None of these isolates were stored, and therefore none were sequenced.

**FIG 2:**
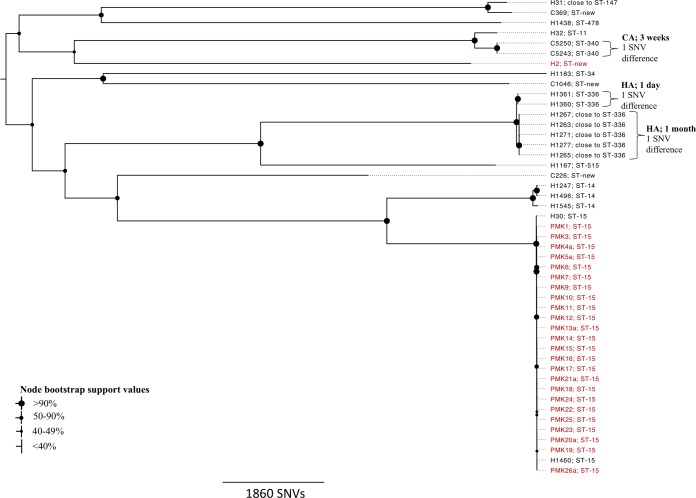
Maximum-likelihood phylogeny of all sequenced Klebsiella pneumoniae study isolates (deduplicated by individual). Isolates in red represent those originally considered part of case clusters on the basis of clinical suspicion and susceptibility testing. Additional closely genetically related clusters of nonoutbreak strains are indicated with curly brackets. “CA” denotes community-associated strains, and “HA” hospital-associated strains; the time interval listed is that spanning the isolation dates of clustered isolates. Symbols indicating bootstrap support at nodes are defined in the key.

The sequenced K. pneumoniae isolates represented 14 STs; six were novel (Fig. 2). The strains exhibited considerable diversity (56,947 core variable sites as mapped to reference strain MGH78578; mean call rate of 87%). H2, part of the epidemiologically defined cluster, was distantly related to the outbreak cases (16,557 SNVs from PMK1). Conversely, H1460, not part of the cluster, was part of the genetically defined outbreak (1 SNV from PMK26a), as was a hospital-associated strain from an adult, H30 (21 SNVs from PMK1). All outbreak isolates were sequence type 15 (ST15). Three additional smaller, closely genetically related clusters were observed; two neonatal hospital-associated clusters and a pair of community-associated infections within 3 weeks of each other (Fig. 2; see also Section 1 in the supplemental material).

**FIG 3:**
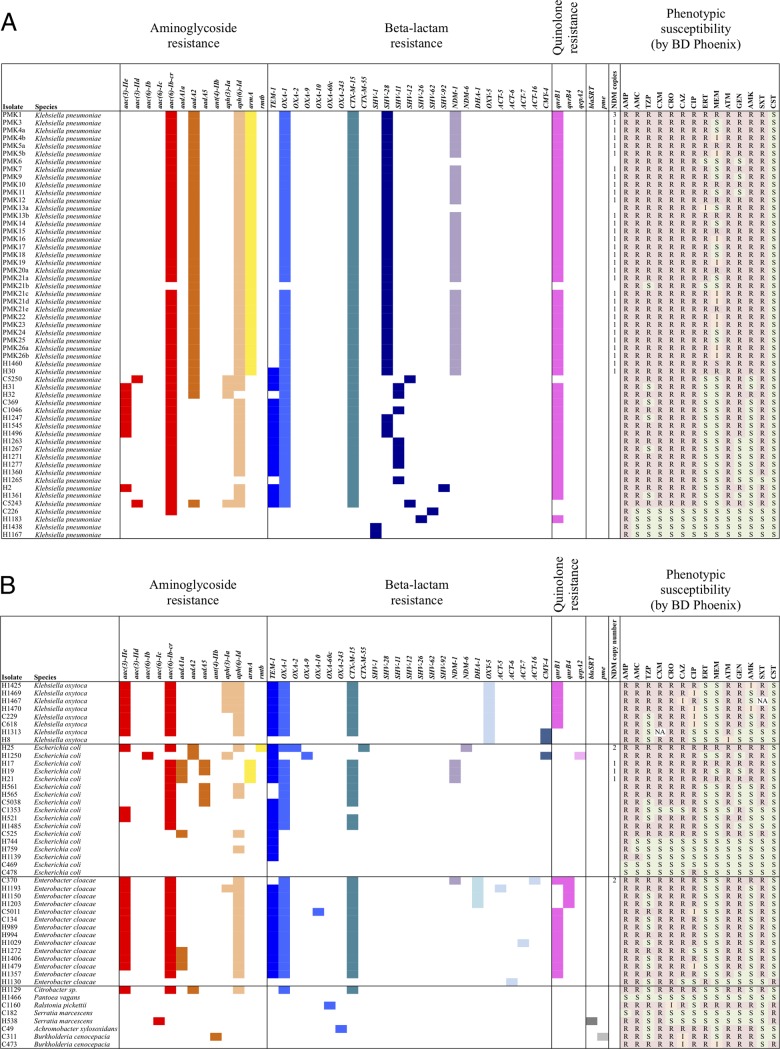
Resistance genotypes and susceptibility phenotypes as determined by the Phoenix automated system are shown for all sequenced Klebsiella pneumoniae isolates (A) and for all sequenced non-Klebsiella pneumoniae isolates (B). Copy numbers calculated for *bla*_NDM_, where present, are also shown. Susceptibility categories were determined by Phoenix in accordance with EUCAST breakpoints. S, susceptible; I, intermediate; R, resistant; NA, no result available; AMP, ampicillin-amoxicillin; AMC, amoxicillin-clavulanate; TZP, piperacillin-tazobactam; CXM, cefuroxime; CRO, ceftriaxone; CAZ, ceftazidime; CIP, ciprofloxacin; ERT, ertapenem; MEM, meropenem; ATM, aztreonam; GEN, gentamicin; AMK, amikacin; SXT, trimethoprim-sulfamethoxazole; CST, colistin.

We also sequenced 47 other clinical isolates representing different species (Fig. 3A and B); the earliest isolate was from 5 January 2008 and the latest from 1 August 2012. Within the wider hospital, several isolates causing bloodstream infections in neonates remained unsequenced, including five K. pneumoniae isolates, four isolates defined as Klebsiella spp., two Escherichia coli isolates, seven Acinetobacter spp. isolates, and two Klebsiella oxytoca isolates. Genotypic and phenotypic multidrug resistance was very common in both hospital- and community-associated isolates: 13/21 (62%) nonoutbreak K. pneumoniae isolates, 12/14 (86%) Enterobacter cloacae isolates, and 6/8 (75%) Klebsiella oxytoca isolates contained at least one variant of each of *aac*, *bla*_TEM_, *bla*_OXA_, *bla*_CTX-M_, and *qnr* in combination. Three E. coli isolates and one E. cloacae isolate contained *bla*_NDM-1_, and a further E. coli isolate contained *bla*_NDM-6_.

### Detailed outbreak strain analysis.

**FIG 4:**
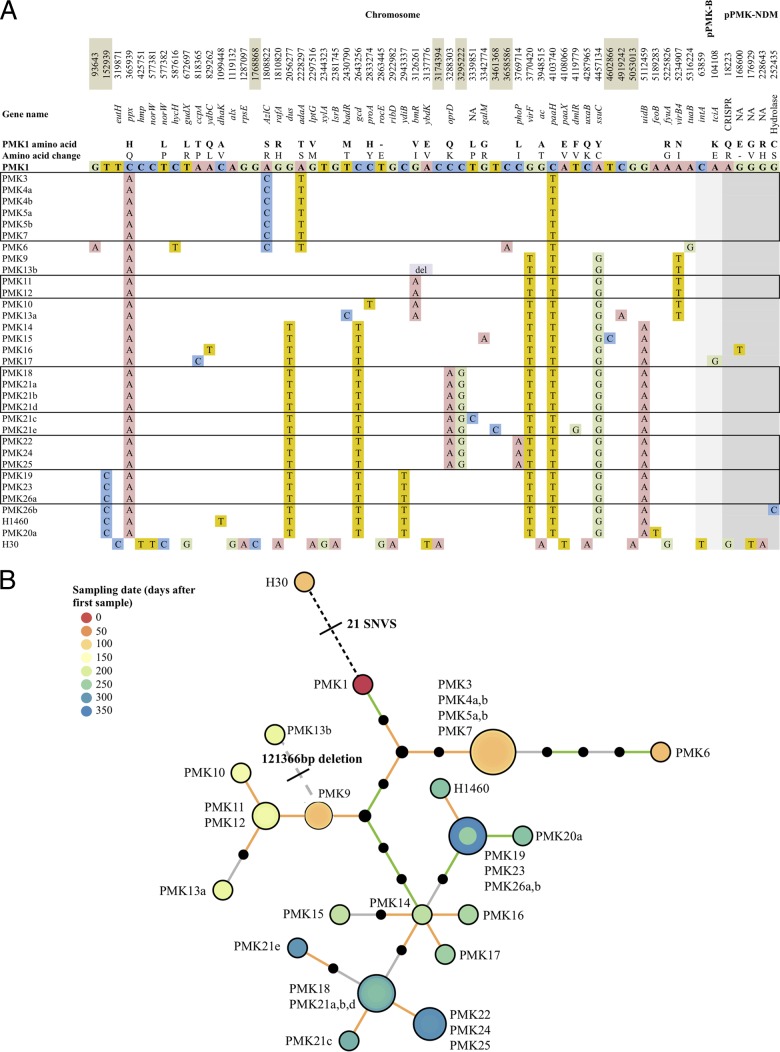
(A) Nucleotide and amino acid level variations in outbreak isolates with respect to the sequences of the PMK1 reference chromosome and pPMK1-B and pPMK1-NDM. del, deletion; NA, unannotated hypothetical protein; -, stop codon. The order of the isolates approximates the outbreak time frame but also accommodates the grouping of isolates that share identical genetic sequences based on mapping to the PMK1 reference chromosome (i.e., 0 SNV differences; shown in boxes with black borders). Brown-shaded chromosomal positions represent noncoding positions, gray-shaded positions represent positions in plasmids. Nonsynonymous, persistent mutations occurred in *ppx* (an exopolyphosphatase), *azlC* (an azaleucine resistance protein), *adaA* (a methyltransferase), *gcd* (a quinoprotein glucose dehydrogenase), *bmR* (a transcriptional repressor), *oprD* (an outer membrane porin), *ssuC* (an alkane sulfonate transporter subunit), and *virB4* (a type IV secretion system). (B) Phylogenetic tree summarizing chromosomal genetic relationships between all outbreak isolates. Colored nodes represent sampled isolates and black nodes unsampled intermediates; node colors are defined in the key. Each solid branch represents a single SNV, with branch colors indicating mutation types as follows: orange, nonsynonymous; green, synonymous; gray, intergenic; gray dashed line, 121,366-bp deletion; black dashed line, 21 single-nucleotide variants (SNVs).

Fifty-three high-confidence chromosomal SNVs were identified during the outbreak (Fig. 4A and B), 21 uniquely in the adult H30 strain. Sixteen SNVs emerged and persisted in more than one isolate, 14 in coding sequences. Of these, eight resulted in nonsynonymous mutations (Fig. 4A). There was a large, 121,366-bp deletion in PMK13b (reference bp positions, 3047928 to 3169294).

From the time-scaled phylogenetic tree (see Section 6 in the supplemental material), the outbreak strain's mutation rate was estimated at 3.65 × 10^−6^ (95% confidence interval [CI], 2.45 × 10^−6^ to 4.89 × 10^−6^) mutations per called site per year, equating to 18.4 (95% CI, 12.3 to 24.6) mutations per genome per year. The time to most recent common ancestor of the adult-associated H30 strain and the PMK1 strain cultured from the first infected neonatal case was estimated at between 1 and 7 months before the neonatal outbreak was observed.

**FIG 5:**
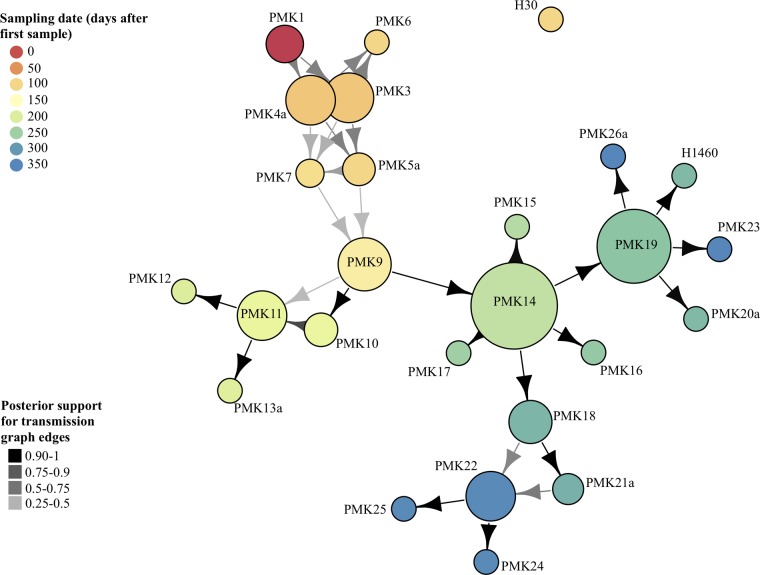
Transmission network inferred using Outbreaker. The sizes of the nodes reflect the numbers of secondary cases per infected individual. The colors of nodes reflect the sampling date as indicated in the key at the upper left. Shading of arrows represents the degree of posterior support for the transmission link, as indicated in the key at the bottom left.

The transmission network inferred by Outbreaker (Fig. 5) demonstrates uncertainty around the specific transmission links for early strains (PMK3 to PMK9). Four individuals harboring these strains shared ward space and time (PMK3 to PMK5 and PMK7), and the network is consistent with direct transmissions. PMK9 may have contaminated the ward or equipment in the established septic nursery or colonized an unsampled asymptomatic host, leading to the infection represented by PMK10 and, possibly, also to PMK11 (although this case was also consistent with a direct transmission from PMK10) and later spreading to the individual harboring PMK14. The link between PMK11 and PMK12 again most likely represents an indirect transmission through either an environmental or asymptomatic source, and the link between PMK11 and PMK13 represents an event across wards. Based on the available epidemiologic data (Fig. 1), the spread from PMK14 most likely occurred within the established septic nursery (possibly directly to PMK15, otherwise indirectly via equipment or a colonized asymptomatic contact). The links between PMK18, PMK21a, PMK22, PMK24, and PMK25 could potentially have been established through sequential transfer from the established septic nursery to the NICU and then to the overflow septic nursery. PMK19 to PMK20 may represent a direct transmission event, but the nature of the link between PMK19 and cases PMK23 and PMK26a is less evident. The model identifies PMK14 and PMK19 as contributing to the largest number of secondary outbreak cases, with H30 excluded from the inferred transmission network.

Four complete plasmid sequences were identified in PMK1. The *bla*_NDM-1_-containing plasmid, pPMK1-NDM (304,526 bp), was a multireplicon (IncHI1B/IncFIB) plasmid with antibiotic resistance determinants that included *aac(6′)-Ib-cr*, *aadA2*, *bla*_CTX-M-15_, *bla*_OXA-1_, *folP*, *catA1*, *dfrA12*, *armA*, and a large conjugative transfer module. A number of mercury resistance (*mer*) genes were present (see Section 9 in the supplemental material).

pPMK1-A (187,571 bp), also a multireplicon (IncFII_K_/IncFIB_K_) and likely a conjugative plasmid, contained tetracycline resistance genes *tetA* and *tetR* and iron (*fec*), arsenic (*ars*), copper (*cop*), tellurite, and silver (*sil*) resistance gene cassettes. pPMK1-B (111,693 bp) was a colE1-type, IncFIB plasmid, containing a tellurite resistance gene but lacking obvious conjugative transfer genes. pPMK1-C (69,947 bp) contained *aph(6)-Id* and *aph(3′)-Ib*-like resistance genes encoding streptomycin and kanamycin/neomycin resistance, respectively (see Section 9 in the supplemental material).

**FIG. 6:**
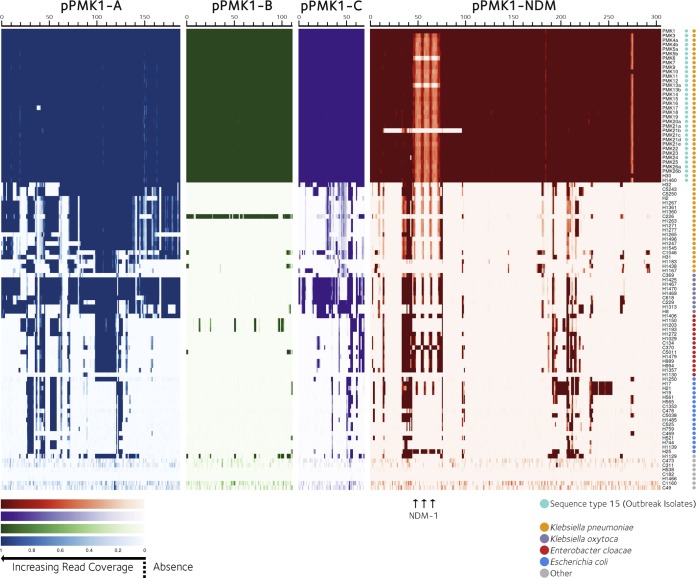
Mapping of coverage of reference plasmids in outbreak and nonoutbreak strains. Reference plasmid coordinates are displayed in kilobases. Heatmap values represent the number of bases mapped, scaled by the number of bases mapped to the whole reference plasmid. A value of zero represents absence. A value of 1 represents presence at the average coverage or greater. Values between zero and 1 represent presence at a lower-than-average coverage.

All four plasmids were highly conserved across the outbreak isolates (Fig. 6). There were five SNVs in the NDM-containing plasmid and two in pPMK-B, with three and one SNV, respectively, in these plasmids only in H30. No SNV-level variation was observed for pPMK-A or pPMK-C (Fig. 4A).

Plasmid pPMK1-NDM contained two regions not present in other outbreak strains. The first was a tandem duplication of the *bla*_NDM-1_ region (kb positions 56 to 76), resulting in three *bla*_NDM-1_ copies, and the second an acquisition of two transposases (bp positions 272471 to 275170) in the plasmid structure. pPMK6-NDM and pPMK13a-NDM shared a 26-kb deletion (bp positions 454501 to 70264), including *bla*_NDM-1_. pPMK21b-NDM contained a larger, 83-kb deletion (bp positions 13135 to 96114) involving *bla*_NDM-1_ and other antibiotic resistance genes, including *aac(6′)-Ib-cr*, *bla*_OXA-1_, and *catB3. bla*_NDM-1_ gene deletions correlated with reversion to ertapenem susceptibility. pPMK24-NDM contained a small, 2,401-bp deletion representing three phage-related open reading frames. The only other variation in plasmid gene presence was a deletion in bp positions 36552 to 40801 of pPMK17-A, involving tetracycline resistance genes (*tetA* and *tetR*).

Comparison of plasmid structures across nonoutbreak Klebsiella strains and non-K. pneumoniae bacteria found outbreak plasmids to be restricted to the outbreak Klebsiella strains (Fig. 6). Partial exceptions were (i) pPMK1-B, large tracts of which were also found in a community-associated K. pneumoniae strain (C226), and (ii) pPMK1-A, regions of which were found in several nosocomial and community-associated K. pneumoniae and K. oxytoca strains. pPMK1-NDM, however, was not observed in any nonoutbreak strains, and in the five other NDM-positive isolates, *bla*_NDM_ was located in non-pPMK1-NDM genetic backgrounds (four were E. coli isolates H17, H19, H21 [closely genetically related; data not shown], and H25, and one was the E. cloacae isolate C370).

## DISCUSSION

Despite the time lapses between isolates and initial uncertainty as to whether all cases were linked, WGS clearly demonstrated that the outbreak was caused by a single, clonal strain of ST15 K. pneumoniae in association with a conserved population of four plasmids, including a *bla*_NDM-1_-containing plasmid. The intervals between case clusters suggest persistence of the NDM-1 K. pneumoniae strain in the unit environment or in asymptomatic carriage, potentially supported by the isolation of Klebsiella spp. from environmental samples and rectal swabs taken from staff. Both environmental contamination and asymptomatic colonization are likely contributors to the transmission of drug-resistant K. pneumoniae in the nosocomial setting (17), although the relative contribution of each route is unknown and could feasibly vary among lineages. The assessment of combined epidemiologic data and the transmission network inferred from sampling dates and the genomic data in this study strongly supports the view that even-wider sampling frames are needed to fully understand the dynamics of these outbreaks, given that both direct and indirect human and environmental transmissions are likely to be occurring. The time-scaled analysis of the outbreak isolates and H30 suggests that an ancestral strain predating PMK1 and H30 was present somewhere between 1 and 7 months before the identification of the first infected neonatal case—this ancestral strain may have been present in the parents, other patients, hospital staff, or the hospital environment.

While it is impossible to exclude repeated introductions of either the strain or the *bla*_NDM-1_-containing plasmid into the pediatric critical care setting from the community or elsewhere in the hospital, the extraordinary degree of similarity between outbreak strains, in contrast to other contemporaneous strains from both locations, makes this less likely. Regarding the selection of the wider set of isolates for sequencing, we specifically avoided characterizing only phenotypically carbapenem-resistant organisms, given the known lack of sensitivity of phenotypic methods in the presence of carbapenemases (12), aiming to determine whether there was any evidence for wider dissemination of the outbreak plasmids or the outbreak K. pneumoniae strain in the absence of *bla*_NDM_. The wider sampling and detailed plasmid analysis are major strengths of our study and expand on the two previous WGS outbreak investigations (16, 17); without such data, uncertainty about transmission versus repeated importations of strains or dissemination of drug resistance plasmids remains, particularly in high-prevalence contexts.

This study exemplifies the potential of using WGS to benefit both outbreak management and antimicrobial treatment. In particular, long-read (30-kb) PacBio sequencing enabled us to produce reference assemblies of the isolate cultured from the first infected neonatal case, including both chromosome and plasmids, which were then used as a comparator for Illumina short-read data sets for the other isolates, allowing fine-scale definition of genetic differences between strains and probabilistic interpretation of likely transmission pathways. As well as resolving the temporally distinct clusters of NDM-1 K. pneumoniae isolates into a single year-long outbreak, phylogenetic analysis identified several other unrecognized clusters of antimicrobial-resistant isolates, indicating both nosocomial (two separate CTX-M-15 K. pneumoniae outbreaks, one NDM-1 E. coli outbreak, and two K. oxytoca and three E. cloacae clusters [data not shown]) and community (CTX-M-15 K. pneumoniae) transmission.

Laboratory susceptibility phenotyping for susceptible, intermediate, and resistant (SIR) categories were inconsistent in some cases between isolates with the same complement of resistance genes, even for the highly genetically related outbreak strains, highlighting the challenges of relying on this for cluster identification (observed for meropenem in the presence of NDM-1 and gentamicin in the presence of *armA*) (Fig. 4A). Although sequence-based susceptibility prediction has yet to be correlated with patient-level clinical outcomes, it appears to be sensitive and specific (22, 32) and indicates the potential future value of WGS in managing patients when current routine laboratory turnaround times are matched. Single-strand sequencing platforms and new, fast genome assemblers (33) are likely to make resistance prediction from clinical specimens, such as blood cultures, possible within hours of sampling.

This NDM K. pneumoniae outbreak terminated after a year, and hospital-wide neonatal critical care deaths almost halved after it ended, consistent with its suppression or eradication. This is of interest for two reasons: First, *bla*_NDM_ is known to be locally prevalent and was found in 5 of 68 (7%) sequenced, nonoutbreak clinical isolates in this study, and second, the hospital is of older construction and potentially less amenable to infection control. Carbapenemase-associated K. pneumoniae outbreaks have been shown to be difficult to control in some settings, even those where resources are less restricted (5). More recently, a separate NDM-1 Enterobacter cloacae outbreak was observed in the hospital, albeit in association with a different plasmid vector (data not shown), and was terminated following fumigation of a number of the clinical units, demonstrating the need for ongoing surveillance and comprehensive infection control.

ST15 K. pneumoniae is one of the dominant global clones, associated with a range of beta-lactamases, including NDM and CTX-M-15 (34, 35). Its success may partly relate to the accumulation of resistance without fitness costs (36). The outbreak strain stably supported several plasmids totaling nearly 700 kb, 13% of the chromosome size. The outbreak plasmids contained resistance genes, plasmid addiction modules, and genes encoding systems protecting host bacteria against bacteriophages or plasmids, which may have contributed to the strain's dominance and stable persistence.

pPMK1-NDM showed substantial homology to pNDM-MAR (JN420336.1), first identified from ST15 K. pneumoniae isolates in Morocco, but it differed from other NDM-containing plasmids (37). The major differences were the presence of additional NDM-1 copies and further resistance genes in pPMK1-NDM (see Section 10 in the supplemental material). Carbapenem exposure affects NDM-1 copy number, and lack of selection pressure can lead to complete gene deletion (38), providing further evidence to support minimizing unnecessary carbapenem therapy.

Our mutation rate estimate for the outbreak K. pneumoniae strain is higher than the mutation rates of other Enterobacteriaceae, such as E. coli (∼1.1/genome/year) (39). There are no published data on K. pneumoniae mutation rates to our knowledge; it is therefore impossible to ascertain whether this strain was adaptable because of hypermutability or whether this is a species-level phenomenon. Mutation rates define the plausible time frame of acquisition events in bacteria and are critical now that genomic data are increasingly being relied upon to refine transmission epidemiology. Some SNVs were intergenic, highlighting the potential additional resolution achieved using the complete mapped genome rather than extended multilocus sequence typing for transmission analyses (40).

There are several limitations to our study. The NDM-1 copy number variation may have been due to variable selection pressure in strain culture and storage. Selective culture would have aided environmental detection of NDM-containing isolates, and the identification of an environmental source or the presence of a susceptible variant of the outbreak clone was unfortunately impossible given the lack of further susceptibility testing on and/or storage of environmental/fecal carriage isolates for sequencing. Routine surveillance of patients admitted to at-risk units would have been ideal but was not possible given the local resource constraints. The wider set of clinical isolates did not contain other ST15 K. pneumoniae bacteria; these were either present at low frequencies and thus unsampled or were not present at all. Despite our wider sampling, we were still unable to identify the source of the outbreak, a limitation which is likely to be overcome when it is possible to implement WGS as a high-resolution, real-time typing method and use it to refine and extend ongoing outbreak investigations.

In summary, this study highlights challenges in managing clusters of resistant K. pneumoniae infections and demonstrates that, despite a lack of obvious transmission pathways, the same strain can persist in hospital environments for months, sporadically causing disease. Whether this is a consequence of particular combinations of host bacterial strains and NDM-containing and/or other plasmids, representing clinically successful entities with a significant impact on patient outcome, is unclear. However, WGS provides a high-resolution mechanism by which the contribution of these different elements can start to be unraveled.

## Supplementary Material

Supplemental material
