# Supplementary material for: Genome Sequencing of an Extended Series of NDM-Producing Klebsiella pneumoniae Isolates from Neonatal Infections in a Nepali Hospital Characterizes the Extent of Community- versus Hospital-Associated Transmission in an Endemic Setting
Source: Antimicrob Agents Chemother. 2014 Dec;58(12):7347–57. doi: 10.1128/AAC.03900-14 (PMC4249533; doi:10.1128/AAC.03900-14)
Supplement: Supplemental material [file supp_58_12_7347__index.html]

Genome Sequencing of an Extended Series of NDM-Producing Klebsiella pneumoniae Isolates from Neonatal Infections in a Nepali Hospital Characterizes the Extent of Community- versus Hospital-Associated Transmission in an Endemic Setting — Supplemental material 

# Genome Sequencing of an Extended Series of NDM-Producing Klebsiella pneumoniae Isolates from Neonatal Infections in a Nepali Hospital Characterizes the Extent of Community- versus Hospital-Associated Transmission in an Endemic Setting

## Supplemental material

**Files in this Data Supplement:**

- Supplemental file 1 -

  Supplemental sections 1 to 10.

  PDF, 1.4M
