# Supplementary material for: Genome Sequencing of an Extended Series of NDM-Producing Klebsiella pneumoniae Isolates from Neonatal Infections in a Nepali Hospital Characterizes the Extent of Community- versus Hospital-Associated Transmission in an Endemic Setting
Source: Antimicrob Agents Chemother. 2014 Dec;58(12):7347–57. doi: 10.1128/AAC.03900-14 (PMC4249533; doi:10.1128/AAC.03900-14)
Supplement: Supplemental material [file AAC.03900-14_zac012143503so1.pdf]

Supplementary Section 1. Study strain accession and epidemiological details (NCBI BioProject id: PRJNA253300). Neonate defined as ≤28 days of age; child >28 days of age but <16 years; adults ≥16 years.

Sample name	Diagnostic laboratory species identification	Sequencing species identification	Sample date	Sample location source	Sample specimen source	Sample patient source	Sequencing accession
PMK1	*Klebsiella pneumoniae*	*K. pneumoniae*	16/08/2011	Hospital	Blood	Neonate	SAMN02885365
PMK3	*K. pneumoniae*	*K. pneumoniae*	20/11/2011	Hospital	Blood	Neonate	SAMN02885389
PMK4a	*K. pneumoniae*	*K. pneumoniae*	20/11/2011	Hospital	Blood	Neonate	SAMN02885390
PMK4b	*K. pneumoniae*	*K. pneumoniae*	10/12/2011	Hospital	Blood	Neonate	SAMN02885391
PMK5a	*K. pneumoniae*	*K. pneumoniae*	23/11/2011	Hospital	Blood	Neonate	SAMN02885392
PMK5b	*K. pneumoniae*	*K. pneumoniae*	27/11/2011	Hospital	Blood	Neonate	SAMN02885393
PMK6	*K. pneumoniae*	*K. pneumoniae*	21/11/2011	Hospital	Blood	Neonate	SAMN02885394
PMK7	*K. pneumoniae*	*K. pneumoniae*	27/11/2011	Hospital	Blood	Neonate	SAMN02885395
PMK9	*K. pneumoniae*	*K. pneumoniae*	20/12/2011	Hospital	Blood	Neonate	SAMN02885396
PMK10	*K. pneumoniae*	*K. pneumoniae*	15/02/2012	Hospital	Blood	Neonate	SAMN02885366
PMK11	*K. pneumoniae*	*K. pneumoniae*	17/02/2012	Hospital	Blood	Neonate	SAMN02885367
PMK12	*K. pneumoniae*	*K. pneumoniae*	02/03/2012	Hospital	Blood	Neonate	SAMN02885368
PMK13a	*K. pneumoniae*	*K. pneumoniae*	27/02/2012	Hospital	Blood	Neonate	SAMN02885369
PMK13b	*K. pneumoniae*	*K. pneumoniae*	29/02/2012	Hospital	Blood	Neonate	SAMN02885370
PMK14	*K. pneumoniae*	*K. pneumoniae*	08/04/2012	Hospital	Blood	Neonate	SAMN02885371
PMK15	*K. pneumoniae*	*K. pneumoniae*	05/04/2012	Hospital	Blood	Neonate	SAMN02885372
PMK16	*K. pneumoniae*	*K. pneumoniae*	22/04/2012	Hospital	Blood	Neonate	SAMN02885373
PMK17	*K. pneumoniae*	*K. pneumoniae*	01/05/2012	Hospital	Blood	Neonate	SAMN02885374
PMK18	*K. pneumoniae*	*K. pneumoniae*	22/05/2012	Hospital	Blood	Neonate	SAMN02885375
PMK19	*K. pneumoniae*	*K. pneumoniae*	08/05/2012	Hospital	Blood	Neonate	SAMN02885376
PMK20a	*K. pneumoniae*	*K. pneumoniae*	19/05/2012	Hospital	Blood	Neonate	SAMN02885377
PMK21a	*K. pneumoniae*	*K. pneumoniae*	25/05/2012	Hospital	Blood	Neonate	SAMN02885378
PMK21b	*K. pneumoniae*	*K. pneumoniae*	28/05/2012	Hospital	Blood	Neonate	SAMN02885379
PMK21c	*K. pneumoniae*	*K. pneumoniae*	31/05/2012	Hospital	Blood	Neonate	SAMN02885380
PMK21d	*K. pneumoniae*	*K. pneumoniae*	10/06/2012	Hospital	Blood	Neonate	SAMN02885381
PMK21e	*K. pneumoniae*	*K. pneumoniae*	19/06/2012	Hospital	Blood	Neonate	SAMN02885382
PMK22	*K. pneumoniae*	*K. pneumoniae*	22/06/2012	Hospital	Blood	Neonate	SAMN02885383
PMK23	*K. pneumoniae*	*K. pneumoniae*	26/06/2012	Hospital	Blood	Neonate	SAMN02885384

PMK24	*K. pneumoniae*	*K. pneumoniae*	24/06/2012	Hospital	Blood	Neonate	SAMN02885385
PMK25	*K. pneumoniae*	*K. pneumoniae*	24/06/2012	Hospital	Blood	Neonate	SAMN02885386
PMK26a	*K. pneumoniae*	*K. pneumoniae*	26/06/2012	Hospital	Blood	Neonate	SAMN02885387
PMK26b	*K. pneumoniae*	*K. pneumoniae*	30/06/2012	Hospital	Blood	Neonate	SAMN02885388
H8	*Klebsiella oxytoca*	*K. oxytoca*	01/12/2011	Hospital	Blood	Neonate	SAMN02885362
H2	*K. pneumoniae*	*K. pneumoniae*	07/11/2011	Hospital	Blood	Neonate	SAMN02885350
H521	*Escherichia coli*	*E. coli*	12/03/2010	Hospital	Blood	Neonate	SAMN02885356
H538	*E. coli*	*Serratia marcescens*	28/03/2010	Hospital	Blood	Neonate	SAMN02885357
H561	*E. coli*	*E. coli*	20/04/2010	Hospital	Blood	Neonate	SAMN02885358
H565	*E. coli*	*E. coli*	20/04/2010	Hospital	Blood	Neonate	SAMN02885359
H744	*E. coli*	*E. coli*	22/07/2010	Hospital	Blood	Neonate	SAMN02885360
H759	*E. coli*	*E. coli*	29/07/2010	Hospital	Blood	Neonate	SAMN02885361
H989	*Enterobacter* sp.	*Enterobacter cloacae*	28/01/2011	Hospital	Blood	Neonate	SAMN02885363
H994	*Enterobacter* sp.	*E. cloacae*	10/02/2011	Hospital	Blood	Neonate	SAMN02885364
H1029	*E. coli*	*E. cloacae*	13/03/2011	Hospital	Blood	Neonate	SAMN02885315
H1130	*Enterobacter* sp.	*E. cloacae*	12/06/2011	Hospital	Blood	Neonate	SAMN02885317
H1129	*Citrobacter* sp.	*Citrobacter* sp.	12/06/2011	Hospital	Blood	Neonate	SAMN02885316
H1139	*E. coli*	*E. coli*	20/06/2011	Hospital	Blood	Neonate	SAMN02885318
H1150	*Enterobacter* sp.	*E. cloacae*	05/07/2011	Hospital	Blood	Neonate	SAMN02885319
H1203	*Enterobacter* sp.	*E. cloacae*	13/08/2011	Hospital	Blood	Neonate	SAMN02885323
H1272	*Enterobacter* sp.	*E. cloacae*	24/10/2011	Hospital	Blood	Neonate	SAMN02885330
H1357	*Enterobacter* sp.	*E. cloacae*	03/02/2012	Hospital	Blood	Neonate	SAMN02885333
H1406	*Enterobacter* sp.	*E. cloacae*	08/04/2012	Hospital	Blood	Neonate	SAMN02885336
H1479	*Enterobacter* sp.	*E. cloacae*	29/04/2012	Hospital	Blood	Neonate	SAMN02885344
H1466	*Enterobacter* sp.	*Pantoea vagans*	27/05/2012	Hospital	Blood	Neonate	SAMN02885340
H1485	*E. coli*	*E. coli*	11/06/2012	Hospital	Blood	Neonate	SAMN02885345
H17	*E. coli*	*E. coli*	14/07/2012	Hospital	Not blood, other sterile site	Adult	SAMN02885348
H19	*E. coli*	*E. coli*	19/07/2012	Hospital	Not blood, other sterile site	Adult	SAMN02885349
H21	*E. coli*	*E. coli*	27/07/2012	Hospital	Not blood, other sterile site	Adult	SAMN02885351
H25	*E. coli*	*E. coli*	01/08/2012	Hospital	Not blood, other sterile site	Adult	SAMN02885352
H30	*Klebsiella pneumoniae*	*K. pneumoniae*	25/10/2011	Hospital	Blood (Medical ICU)	Adult	SAMN02885353
H31	*K. pneumoniae*	*K. pneumoniae*	11/07/2012	Hospital	Sputum (Medical ward)	Adult	SAMN02885354

H32	*K. pneumoniae*	*K. pneumoniae*	18/07/2012	Hospital	Urine (Gynaecology ward)	Adult	SAMN02885355
H1193	*K. pneumoniae*	*E. cloacae*	03/08/2011	Hospital	Blood	Neonate	SAMN02885322
H1247	*K. pneumoniae*	*K. pneumoniae*	26/09/2011	Hospital	Blood	Neonate	SAMN02885324
H1250	*Klebsiella* sp.	*E. coli*	28/09/2011	Hospital	Blood	Neonate	SAMN02885325
H1263	*Klebsiella* sp.	*K. pneumoniae*	12/10/2011	Hospital	Blood	Neonate	SAMN02885326
H1265	*Klebsiella* sp.	*K. pneumoniae*	13/10/2011	Hospital	Blood	Neonate	SAMN02885327
H1267	*K. pneumoniae*	*K. pneumoniae*	16/10/2011	Hospital	Blood	Neonate	SAMN02885328
H1271	*Klebsiella* sp.	*K. pneumoniae*	21/10/2011	Hospital	Blood	Neonate	SAMN02885329
H1277	*Klebsiella* sp.	*K. pneumoniae*	24/10/2011	Hospital	Blood	Neonate	SAMN02885331
H1360	*K. pneumoniae*	*K. pneumoniae*	04/04/2012	Hospital	Blood	Neonate	SAMN02885334
H1361	*K. pneumoniae*	*K. pneumoniae*	05/04/2012	Hospital	Blood	Neonate	SAMN02885335
H1460	*K. pneumoniae*	*K. pneumoniae*	19/05/2012	Hospital	Blood	Neonate	SAMN02885339
H1467	*K. oxytoca*	*K. oxytoca*	24/05/2012	Hospital	Blood	Neonate	SAMN02885341
H1470	*K. oxytoca*	*K. oxytoca*	26/05/2012	Hospital	Blood	Neonate	SAMN02885343
H1496	*K. pneumoniae*	*K. pneumoniae*	12/07/2012	Hospital	Blood	Neonate	SAMN02885346
H1545	*K. pneumoniae*	*K. pneumoniae*	27/07/2012	Hospital	Blood	Neonate	SAMN02885347
H1425	*K. oxytoca*	*K. oxytoca*	15/04/2012	Hospital	Blood	Neonate	SAMN02885337
H1167	*Klebsiella* sp.	*K. pneumoniae*	05/07/2011	Hospital	Blood	Neonate	SAMN02885320
H1183	*K. pneumoniae*	*K. pneumoniae*	17/07/2011	Hospital	Blood	Neonate	SAMN02885321
H1313	*K. oxytoca*	*K. oxytoca*	04/12/2011	Hospital	Blood	Neonate	SAMN02885332
H1438	*K. pneumoniae*	*K. pneumoniae*	01/05/2012	Hospital	Blood	Neonate	SAMN02885338
H1469	*K. oxytoca*	*K. oxytoca*	21/05/2012	Hospital	Blood	Neonate	SAMN02885342
C5011	*Enterobacter* sp.	*E. cloacae*	05/01/2008	Community	Blood or urine	Child	SAMN02885309
C5038	*E. coli*	*E. coli*	09/02/2008	Community	Blood or urine	Child	SAMN02885310
C5243	*Klebsiella* sp.	*K. pneumoniae*	07/09/2008	Community	Blood or urine	Child	SAMN02885311
C5250	*Klebsiella* sp.	*K. pneumoniae*	25/09/2008	Community	Blood or urine	Child	SAMN02885313
C49	*Pseudomonas* sp.	*Achromobacter xylosoxidans*	26/02/2009	Community	Blood or urine	Child	SAMN02885308
C134	*Enterobacter* sp.	*E. cloacae*	12/05/2009	Community	Blood or urine	Child	SAMN02885297
C182	*Enterobacter* sp.	*S. marcescens*	06/06/2009	Community	Blood or urine	Child	SAMN02885299
C226	*Klebsiella* sp.	*K. pneumoniae*	16/06/2009	Community	Blood or urine	Child	SAMN02885300
C229	*Klebsiella* sp.	*K. oxytoca*	16/06/2009	Community	Blood or urine	Child	SAMN02885301
C311	*Pseudomonas* sp.	*Burkholderia cenocepacia*	20/08/2009	Community	Blood or urine	Child	SAMN02885302

C370	*Enterobacter* sp.	*E. cloacae*	21/10/2009	Community	Blood or urine	Child	SAMN02885304
C369	*Klebsiella* sp.	*K. pneumoniae*	21/10/2009	Community	Blood or urine	Child	SAMN02885303
C473	*Pseudomonas* sp.	*B. cenocepacia*	25/12/2009	Community	Blood or urine	Child	SAMN02885306
C469	*E. coli*	*E. coli*	27/12/2009	Community	Blood or urine	Child	SAMN02885305
C478	*E. coli*	*E. coli*	30/12/2009	Community	Blood or urine	Child	SAMN02885307
C525	*E. coli*	*E. coli*	11/03/2010	Community	Blood or urine	Child	SAMN02885312
C618	*Klebsiella* sp.	*K. oxytoca*	10/05/2010	Community	Blood or urine	Child	SAMN02885314
C1046	*K. pneumoniae*	*K. pneumoniae*	06/04/2011	Community	Blood or urine	Child	SAMN02885295
C1160	*Pseudomonas* sp.	*Ralstonia pickettii*	07/07/2011	Community	Blood or urine	Child	SAMN02885296
C1353	*E. coli*	*E. coli*	29/01/2012	Community	Blood or urine	Child	SAMN02885298

Supplementary Section 2. **Infection control measures implemented in response to the case clusters**

1. 1% Virkon to clean equipment; previously, Savlon (0.3g chlorhexidine gluconate and 3g cetrimide/100mLs; diluted 1:30 in 70% alcohol)
2. Surface cleaning of floors, walls, ceilings, furniture and medical equipment following the identification of each new case.
3. Fumigation of the neonatal units on 02/Nov/2011 and 15/Dec/2011.
4. Inspection of and chlorination of water sources (27/Nov/2011)
5. Water purification by reverse osmosis and the use of purified water in ventilator humidifiers
6. Paper towels to replace cloth towels in all neonatal units
7. Routine change of staff and visitor gowns every morning
8. Hand washing implemented for all visitors; importance of hand hygiene emphasized to all staff on the neonatal units
9. Use of disposable tubing for ventilators
10. Cleaning protocols for ventilators implemented
11. Bedside Chlorhexidine hand cleansing solution (Microshield) used deployed in all neonatal units (equivalent to 0.5% w/v chlorhexidine gluconate + 70% ethanol v/v)
12. Implementation of enhanced microbiological surveillance
13. Closure of original clean nursery and repair of a leaking toilet in the ward above, which was being used by women undergoing vesico-vaginal fistula repair surgery.

Supplementary Section 3. Details of species identification using Kraken

Illumina reads from each isolate were compared to the complete bacterial, archaeal, and viral genomes in RefSeq (as of March 30, 2014); for each sample the species level match with the highest number of hits was reported.

Supplementary Section 4. List of reference genomes used for mapping

Species	Reference for mapping
E. coli	CFT073; GenBank: AE014075.1
Klebsiella pneumoniae subsp. *pneumoniae*	MGH78578; GenBank: CP000647.1
Klebsiella oxytoca	E718; RefSeq: NC_018106.1
Enterobacter cloacae subsp. *cloacae*	ATCC 13047; RefSeq: CP001918.1
Pseudomonas aeruginosa	PA01; RefSeq: NC_002516.2
Serratia marcescens	WW4; RefSeq: NC_020211
Pantoea sp.	At9b ; RefSeq: NC_014837.1

Supplementary Section 5. Details of phyML model

We used PhyML version 3.0, with a generalized time-reversible (GTR) nucleotide substitution model, a gamma-distribution with four rate categories to estimate among-site variation in substitution rates, and 100 bootstrap replicates. Sites where at least one sample had a null/missing call were excluded from the input. For the input alignment, the variant sites derived from mapping to the MGH78578 reference were "padded" with invariant sites in a proportion consistent with the GC content and length of the reference genome (5.69Mb, 57.1%GC content).

Supplementary Section 6. Details of BEAST analysis, and sites excluded from the analysis
Three separate runs on the dataset were undertaken using a strict molecular clock model with the following priors: (i) a GTR nucleotide substitution model with estimated base frequencies; (ii) a discrete gamma distribution with four categories to account for variable substitution rates at each site; (iii) a constant population size; (iv) a random starting tree; and (v) a Monte Carlo Markov Chain (MCMC) length of 30000000 with sampling logged every 1000 iterations. The output of the three runs with respect to mixing and convergence was compared using Tracer v1.5 [33]; good mixing and convergence were observed and effective sampling sizes for all parameters were above 300. Log and tree files for the respective runs were combined with down-sampling using LogCombiner v1.7.5; mutation rates and the phylogeny were determined from these. TreeAnnotator v1.7.5 was used to select the maximum clade credibility tree. The figure below represents the BEAST phylogeny. Colored isolates represent longitudinally sampled isolates from the same individual/color. Blue bars around the node represent the 95% credibility interval around the node height, and in this time-scaled context, the uncertainty around the time-to-most-recent-common-ancestor (TMRCA). Starred nodes have posterior support values >98%.

For the input alignment, 51 variant sites derived from mapping to the Pacbio-derived chromosomal reference for the outbreak strain (excluding positions 3126261 and 3137776 which had been affected by the large deletion in PMK13b) were "padded" with invariant sites (in proportions consistent with the ACGT content of reference chromosome) to the length of the called genome (5,038,898/5,317,001 bases; represents sites where bases were called in all sequences). The molecular clock generated by BEAST was then multiplied by the called genome length to give a mutation rate/genome/year.

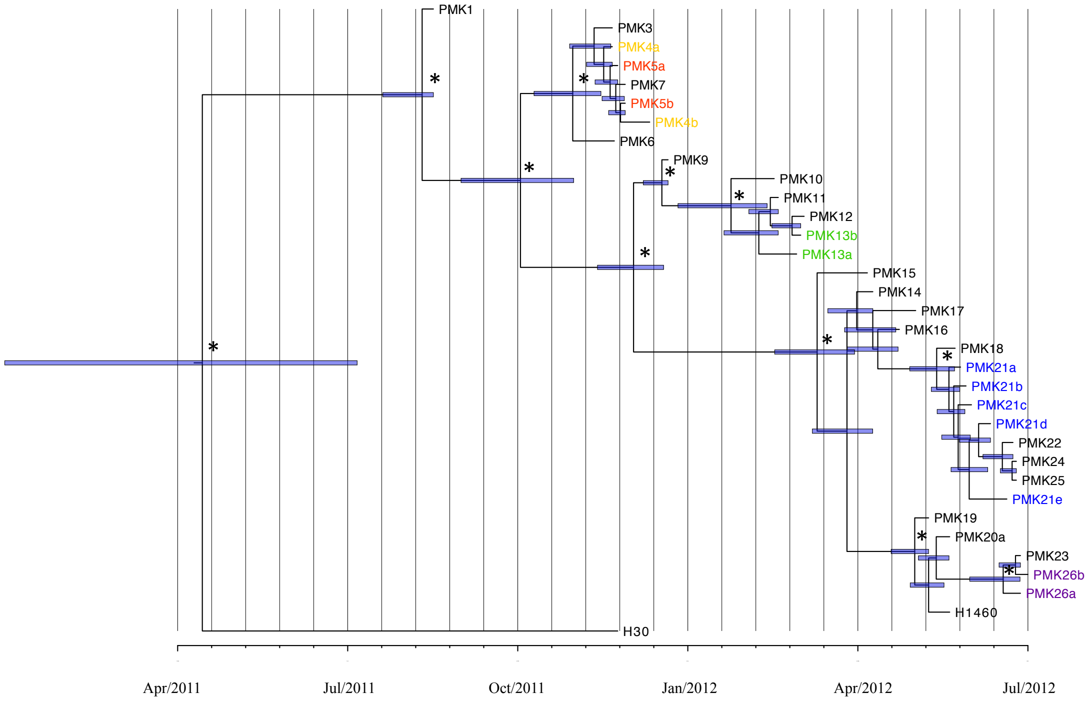

Supplementary Section 7. Details of Outbreaker analysis

An alignment of the reference sequence with inserted SNVs for each of the first isolates obtained from each outbreak case was uploaded into R. The probability distribution of the generation time (the time between colonization of a primary case and transmission to a secondary case) was set up to follow an exponential decline, with the highest probability for transmission estimated for cases closer in time to the primary case. A model with a mean generation time of 50 days was selected, and four parallel MCMC runs were performed for 30,000,000 iterations. Good convergence on visualization of the trace of log-posterior values was observed (3,000,000 iterations were removed as burn-in).

Posterior support for the edges of the transmission tree and estimated mean times to infection were obtained from the posterior ancestries ("alpha" columns in the outbreaker MCMC output) and the dates of infection ("Tinf" columns in the outbreaker MCMC output). The posterior distribution of R values (the R value being the number of secondary cases per infected individual), used to size the nodes in the transmission graph were obtained using the get.R function in the package.

Supplementary Section 8. Details of environmental sampling results

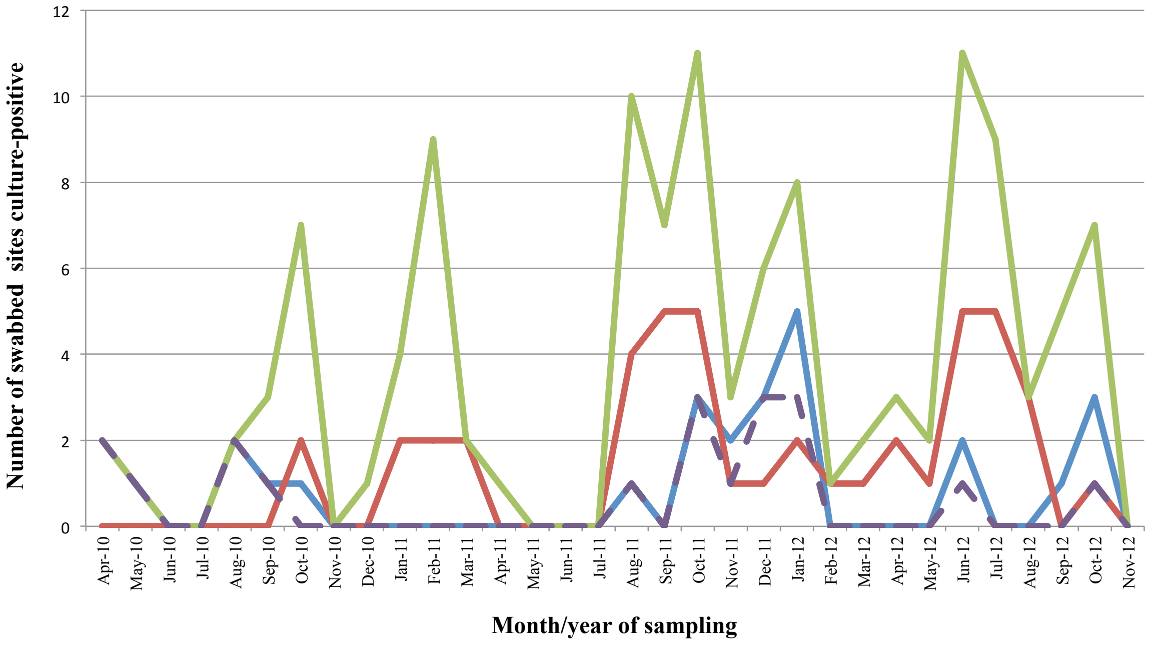

Total number of sites positive
Sites positive for Enterobacteriaceae
Sites positive for *Klebsiella* spp.
Sites positive for *Pseudomonas* spp. or *Acinetobacter* spp.

<u>**Species cultured**</u>

Klebsiella spp.
Escherichia coli
Enterobacter spp.
Citrobacter spp.
Yeasts
Moulds
Micrococcus spp.
Non-haemolytic streptococci
Bacillus spp.
Coagulase negative staphylococci
Staphylococcus aureus
Acinetobacter spp.
Pseudomonas spp.

Supplementary Section 9. Annotated figures of outbreak plasmids

pPMK1-NDM (304526bp)

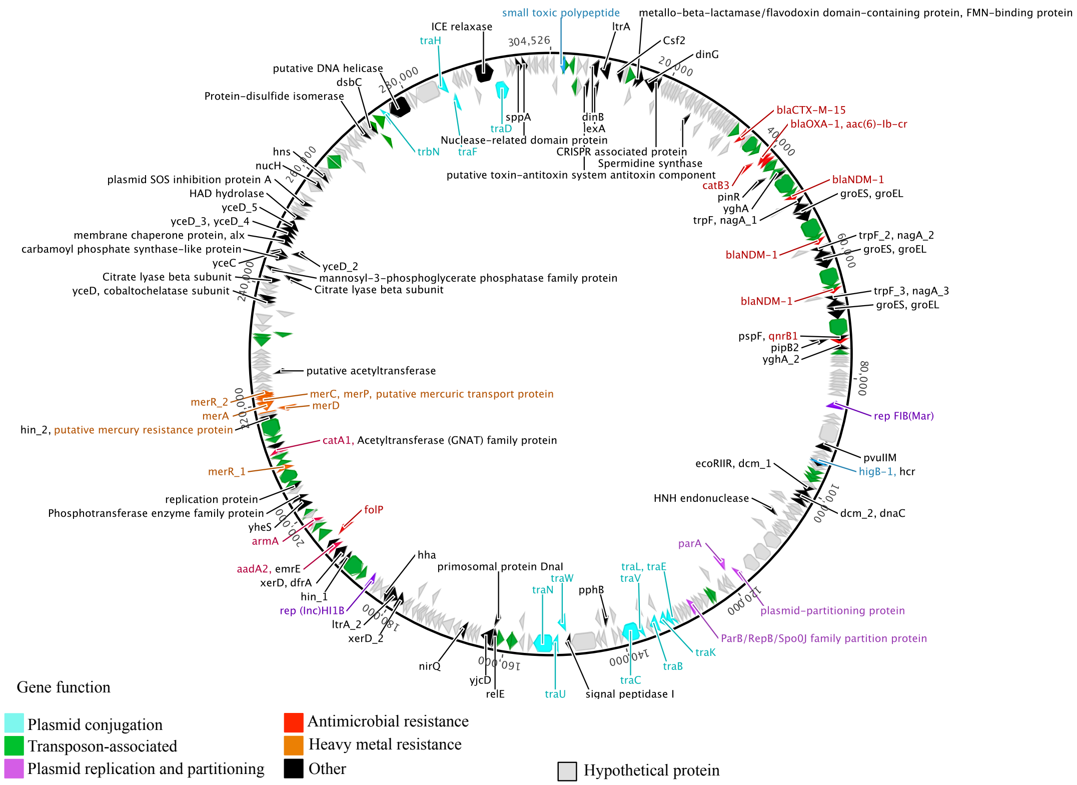

pPMK1-A (187571bp)

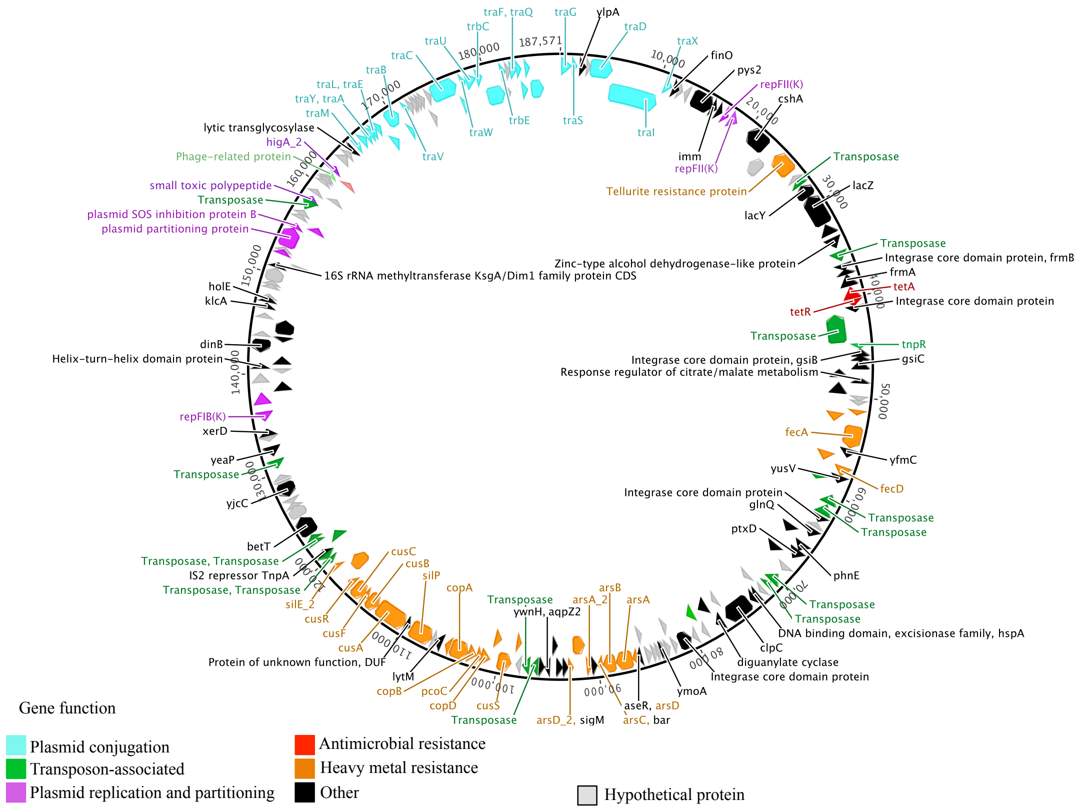

pPMK1-B (111693bp)

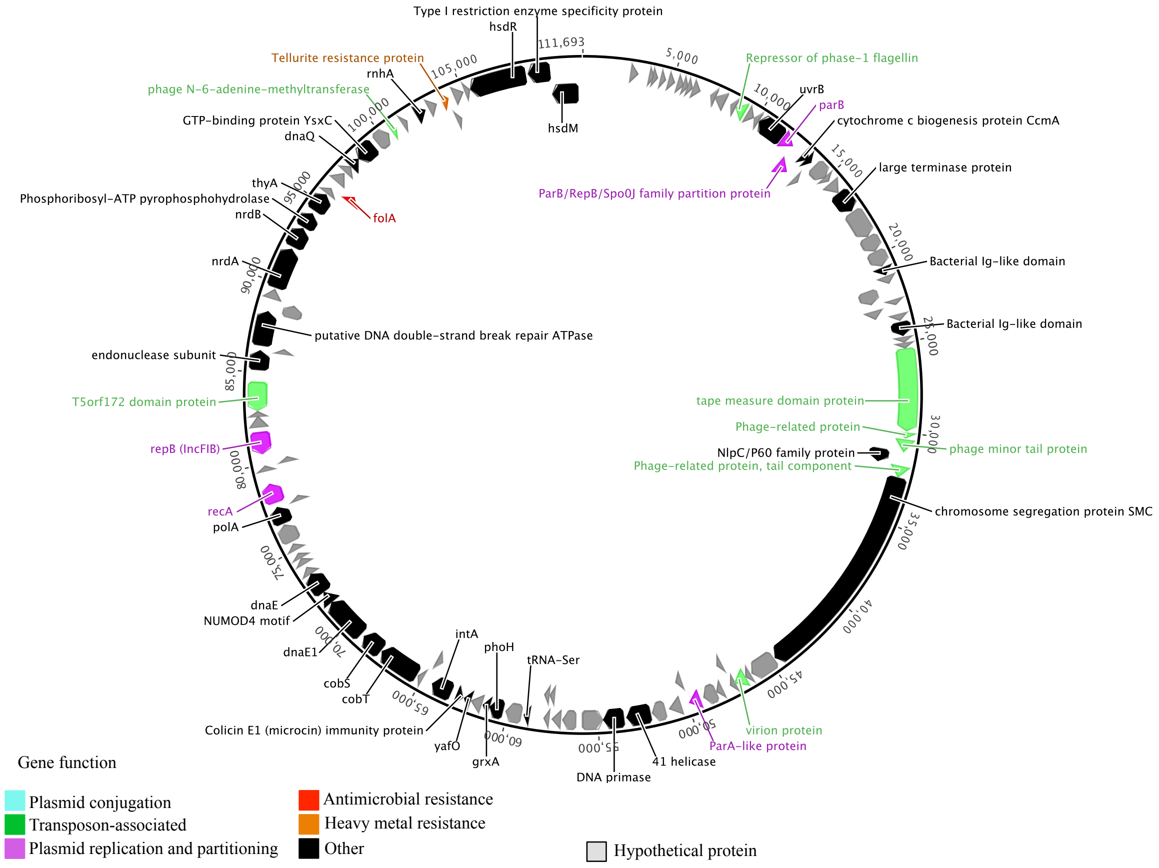

pPMK1-C (69947bp)

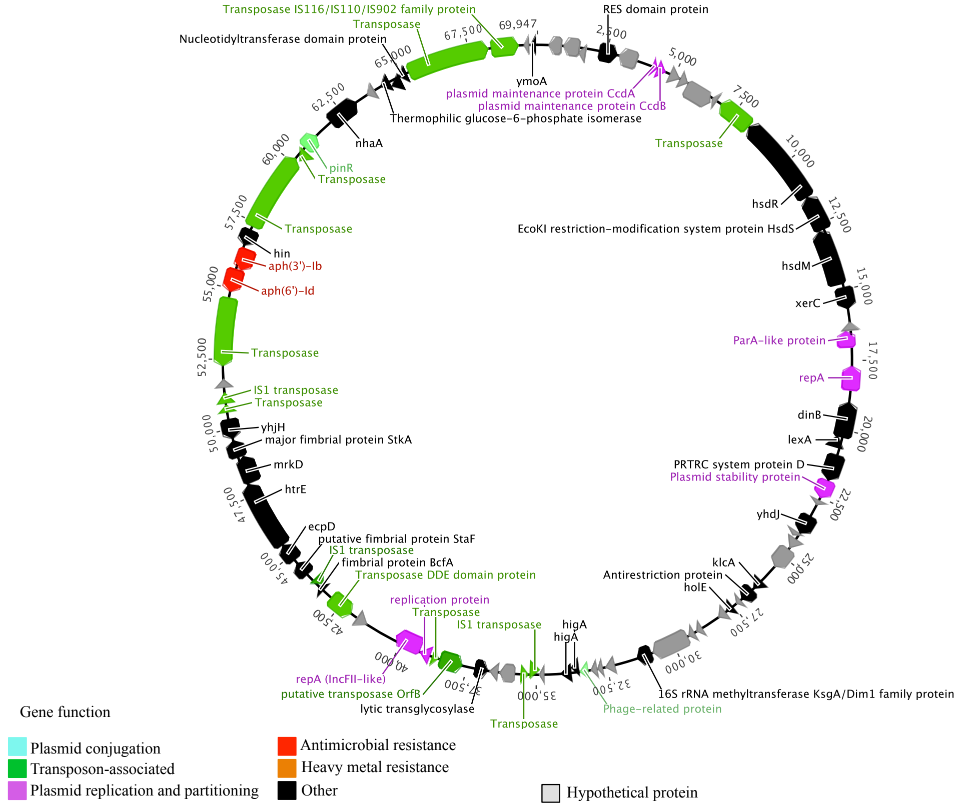

Supplementary Section 10. Structural variation between pNDM-MAR and pPMK1-NDM

Variants in pPMK1-NDM compared to pNDM-MAR			
Start (bp)	End (bp)	Size (bp)	Variant
8388	10297	1909	Insertion
10721	11025	304	Insertion
42490	42872	382	Insertion
43687	61536	17849	Insertion
159099	161364	2265	Insertion
169991	170064	73	Insertion
183046	183886	840	Insertion
191879	205678	13799	Insertion
221706	221721	15	Insertion
263645	265247	1602	Insertion
272471	275179	2708	Insertion
210687	213021	2334	Inversion

Variants in pNDM-MAR compared to pPMK1-NDM			
Start (bp)	End (bp)	Size (bp)	Variant
101532	101734	202	Insertion
180018	180059	41	Insertion
196096	199335	3239	Insertion
174456	176790	2334	Inversion
